# Methylene Blue-Based Combination Therapy with Amodiaquine Prevents Severe Malaria in an Experimental Rodent Model

**DOI:** 10.3390/pharmaceutics14102031

**Published:** 2022-09-24

**Authors:** Jérôme Dormoi, Rémy Amalvict, Mathieu Gendrot, Bruno Pradines

**Affiliations:** 1 Unité Parasitologie et Entomologie, Département Microbiologie et Maladies Infectieuses, Institut de Recherche Biomédicale des Armées, 13005 Marseille, France; 2 Aix Marseille Univ, IRD, SSA, AP-HM, VITROME, 13005 Marseille, France; 3 IHU Méditerranée Infection, 13005 Marseille, France; 4 Centre National de Référence du Paludisme, 13005 Marseille, France

**Keywords:** malaria, *Plasmodium berghei*, antimalarial drug, resistance, in vivo, artemisinin, methylene blue

## Abstract

Untreated malaria can progress rapidly to severe forms (<24 h). Moreover, resistance to antimalarial drugs is a threat to global efforts to protect people from malaria. Given this, it is clear that new chemotherapy must be developed. We contribute new data about using methylene blue (MB) to cure malaria and cerebral malaria in a combined therapy with common antimalarial drugs, including mefloquine (MQ) and amodiaquine (AQ). A C57BL6/J mouse model was used in an experimental cerebral malaria model. Mice were infected with *Plasmodium berghei* ANKA on Day 0 (D0) and the treatment started on D3 (nearly 1% parasitaemia) with AQ, MQ or MB alone or in combination with AQ or MQ. AQ, MQ and MB alone were unable to prevent cerebral malaria as part of a late chemotherapy. MB-based combination therapies were efficient even if treatment began at a late stage. We found a significant difference in survival rate (*p* < 0.0001) between MBAQ and the untreated group, but also with the AQ (*p* = 0.0024) and MB groups (*p* < 0.0001). All the infected mice treated with MB in combination with AQ were protected from cerebral malaria. Partial protection was demonstrated with MB associated with MQ. In this group, a significant difference was found between MBMQ and the untreated group (*p* < 0.0001), MQ (*p* = 0.0079) and MB (*p* = 0.0039). MB associated with AQ would be a good candidate for preventing cerebral malaria.

## 1. Introduction

Malaria remains a public health problem to this day. The infection has an obvious human impact but also a social and economic impact for the different countries affected by this disease [1]. According to the latest World Health Organization (WHO) data, 241 million cases of malaria and 627,000 deaths due to malaria were estimated in 2020 [2]. Almost 55% of all malaria cases globally were concentrated in six countries in 2020 (Angola, Burkina Faso, Democratic Republic of Congo, Mozambique, Nigeria and Uganda). Globally, 602,000 deaths (96% of the global deaths) from malaria were estimated in Africa and 77% of these deaths concerned children under five years old. Malaria cases and deaths increased by 6% (from 227 million to 241 million) and 12% (from 558,000 to 627,000) compared with 2019.

*P. falciparum* drug resistance to most antimalarials emerged in Southeast Asia and has spread to Africa [3]. The latest artemisinin-based therapies also experience therapeutic failures or cases of resistance [4,5,6,7]. This resistance is not only due to a natural evolution of the parasites but also to misuses related to cultural and educational contexts [8,9] or to counterfeit medicines.

It is clear that new curative solutions must therefore be developed. In this context, we propose to evaluate the in vivo efficacy of methylene blue (MB) in combination with standard antimalarial drugs in an experimental rodent malaria model. In its oldest formulation, MB has been known since the end of the 19th century [10,11]. Until about twenty years, MB chemical synthesis was associated with toxic heavy metals and inorganic impurities. This is no longer the case; MB is now produced without heavy metals and impurities thanks to an innovative synthesis process (international patent PCT reference PCT/FR/2007/001193). MB, which is mainly used to treat methemoglobinemia [12,13,14], is also known for its antimalarial properties. MB showed potent in vitro activity in the nanomolar range against *Plasmodium falciparum* [15,16,17,18,19,20,21,22] and *P. vivax* [23,24]. MB had also demonstrated gametocidal action in vitro [25,26,27,28,29] and in vivo [30,31,32]. MB has also demonstrated a protective effect against cerebral malaria in a murine model infected with *P. berghei* [33,34,35]. Moreover, MB showed synergistic effects in vitro and in vivo with dihydroartemisinin [33,36]. MB exerted in vitro additive or synergistic effects in combination with amodiaquine (AQ) and mefloquine (MQ) [36]. However, these two combinations have not been yet evaluated in an experimental rodent model.

Cerebral malaria is a major cause of malaria death. The validity of the experimental cerebral malaria (ECM) model in mice has been shown previously [37,38,39,40,41,42]. The model of *P. berghei* ANKA infection in C57BL/6 mice develops clinical symptoms of cerebral malaria including ataxia, paralysis and coma with death in 8 to 10 days, and high parasite biomass. Many similarities have been described between neurological symptoms due to the *P. berghei* ANKA strain in murin model and *P. falciparum* in human [40,43,44,45].

In this study, we evaluated the potential activity of MB associated with MQ or with AQ. A dose of 5 mg/kg for three days was selected for AQ and MQ, taking into consideration previous studies in malaria mice models [46]. Meanwhile, a dose of 5 mg/kg for five days was used, in light of our previous studies [33,35]. Because MQ has side effects on the central nervous system [47,48,49], this assay aimed to clarify the efficiency of a low dose of MQ combined with MB. In both cases (AQ and MQ), these two antimalarial drugs have long half lives. AQ has a short elimination half-life (approximately five hours) but its main metabolite desethylamodiaquine (DQ) is slowly eliminated with a terminal half-life of 9–18 days [50,51] and MQ is an anti-malarial drug with a 21 days elimination half-life [52,53] in humans.

## 2. Materials and Methods

### 2.1. Experimental Cerebral Malaria (ECM)

All animal experiments were carried out in accordance with EU Directive 2010/63/EU for animal experiments. Fifty female mice were used for therapeutic experiments as previously recommended [54]. Four mice were infected with the murine malaria strain (donor mice) and the blood was used to infect the experimental groups (therapeutic n = 46) with 10^6^ infected red blood cells in 200 µL of filtered normal saline solution (Sodium chloride S9888, Sigma Aldrich, Saint Quentin Fallavier, France). The mice were randomly split between the different groups; eight mice were used in each therapeutic group and six mice were used in the control group. The results of the study were not influenced by sex of mice, as previously documented [55,56].

Mice were housed in a 1290D box (Technoplast, Lyon, France) respecting a minimal volume for each mouse (840 cm^3^). Female C57BL/6J mice (aged 7–8 weeks old) from the Charles Rivers Laboratory (Saint Germain Nuelles, France) were housed under standard and controlled constant laboratory conditions (19–22 °C, relative humidity around 60%), and were fed a normal diet (FT-SAFE-A04, Safe, Augy, France) with water ad libitum. Mice were monitored for general health, weight loss and anaemia throughout infections to ensure they did not reach IACUC endpoints. Shelters and elements to gnaw were provided (JBM022, JBS021, JCEHORA500, Serlab, Montataire, France), and tube handling (JMTT3322, Serlab) was used to manipulate mice and change mice from one box to another following the NR3C recommendations, “How to pick up a mouse”.

The *Plasmodium berghei* ANKA chloroquine-susceptible strain, which was used to infect the mice, was kindly provided by the French National Museum of Natural History (P. Grellier and D. Depoix, Microorganism Communication and Adaptation Molecules (MCAM)—UMR 7245, MNHN, Paris, France).

Experimental cerebral malaria (ECM) was diagnosed by clinical signs based on a modified SHIRPA protocol supplemented by the rapid murine cerebral behaviour scale (RMCBS) [57,58] and a combined scoring method to assess behaviour and welfare using the Morton score grid [59]. Parameters were scored from 0 to 4. A score below 4 correlates with a normal clinical status. We designed our score grid to reflect the fact that an increase in symptoms of ECM in a murine model would increase the score.

All procedures were approved by the Animal Welfare Advisory Committee of IHU Méditerranée Infection and the Animal Research Ethics National Committee (C2EA-14, registration number 2018091915173851-V3).

### 2.2. Treatment Protocol

**Figure 1:**
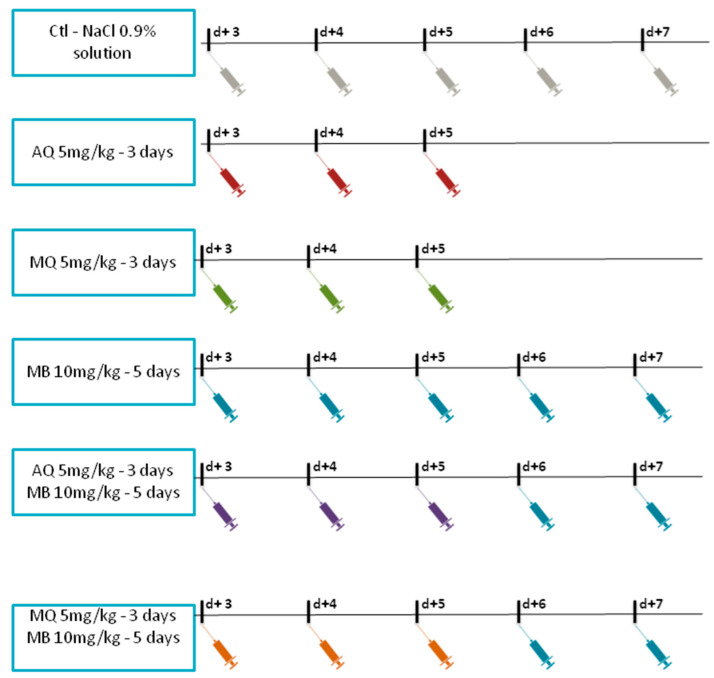
Schemes for malaria mice therapy. Mice were treated from D + 3 to D + 7 for CTL (vehicle), for MB 10 mg/kg alone or in combination with AQ 5 mg/kg or MQ 5 mg/kg, or from D + 3 to D + 5 for AQ 5 mg/kg or MQ 5 mg/kg alone. At D0, mice were infected with *Plasmodium berghei* ANKA.

Each day, treatment was randomly carried out to decrease operator biases. All antimalarial drugs were dissolved in filtered NaCl 0.9% solution with 5% absolute ethanol (*v*/*v*) (ref 32205-1, Sigma Aldrich) and each mouse received 200 µL of solution by intraperitoneal (ip) injection. The mice were first treated when the parasitemia reached at least 1%. AQ hydrochloride (ref 1031004) and MQ hydrochloride (ref M0253000) were purchased from Sigma-Aldrich. A stock solution was prepared and used each day. Solutions were all frozen and thawed before injection, given the stability of common antimalarial drugs [60,61]. MB (methylthioninium chloride 5 mg/mL, Proveblue^®^) was kindly provided by Provepharm SAS (Marseille, France). MB was injected in a normal saline solution with 5% ethanol by ip route at 10 mg/kg for five days. This dilution was used because no interaction or precipitation between these two molecules has been documented. The only effect of this association is the increase in the metabolism of ethanol to CO_2_ in isolated hepatocytes and in intact rats [62,63]. MQ and AQ were delivered by ip in a normal saline solution with 5% ethanol at 5 mg/kg for three days. The group of mouse control received an ip injection of normal saline solution with 5% ethanol (*v*/*v*). The malaria mice therapy diagram is illustrated in Figure 1.

### 2.3. Clinical Follow Up

Mice were observed daily to monitor animal welfare. Every two days, parasitemia was determined using May-Grünwald Giemsa type panoptic colouring (RAL Diagnostics, MCDh reagents, Martillac, France) to stain thin blood smears collected from the tail vein. The number of infected red blood cells per 3000 erythrocytes if >1% and per 10,000 erythrocytes if < 1% of parasitemia. Weight was evaluated each day using a scale.

### 2.4. Statistical Analysis

Statistical analyses were performed with GraphPad Prism v7.05 (GraphPad Software, San Diego, CA, USA). Parasitemia between groups under different treatment conditions were analyzed using one way-ANOVA and the Kruskal–Wallis test. Parasitemia was analyzed, and, when appropriate, the significance of the differences between mean values of two groups was determined using Welsh’s *t*-test. Survival analysis was assessed using the Log-rank (Mantel-Cox) test. Statistical differences in ECM scores between the two groups were assessed using the unpaired test with Welch’s correction.

## 3. Results

### 3.1. Control Group

**Figure 2:**
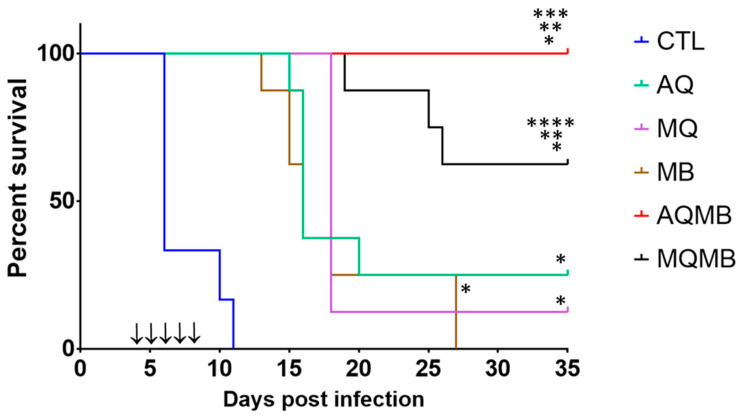
Survival rate for all the therapeutic groups. In blue, control (CTL) with saline solution for five days. In green, AQ 5 mg/kg for three days. In purple, MQ with 5 mg/kg for three days. In brown, MB with 10 mg/kg for five days. In red, MB with 10 mg/kg for five days associated with AQ 5 mg/kg for three days (AQMB). In black, MB with 10 mg/kg for five days associated with MQ 5 mg/kg for three days (MQMB). Black arrows indicate days of treatment. * = significant versus CTL, ** = significant versus MB, *** = significant versus AQ, **** = significant versus MQ.

**Figure 3:**
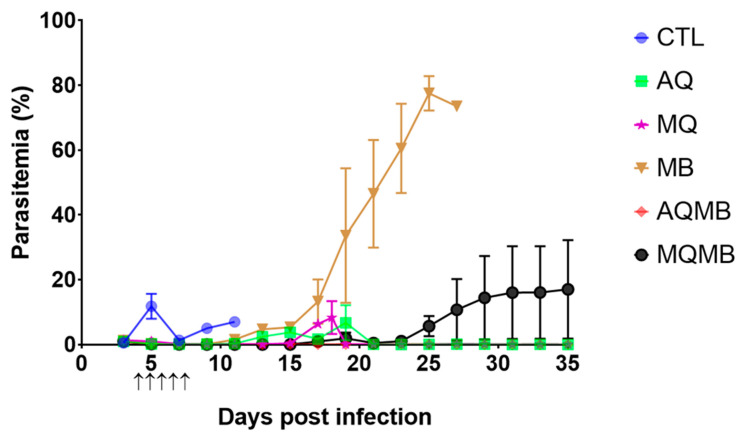
Parasitemia evolution for all the therapeutic groups. In blue, control (CTL) with saline solution for five days. In green, AQ 5 mg/kg for three days. In purple, MQ with 5 mg/kg for three days. In brown, MB with 10 mg/kg for five days. In red, MB with 10 mg/kg for five days associated with AQ 5 mg/kg for three days (AQMB). In black, MB with 10 mg/kg for five days associated with MQ 5 mg/kg for three days (MQMB). Black arrows indicate days of treatment.

In the control (CTL) group, all mice died between D6 and D11 (Figure 2 and Figure 3). Half of the mice experienced severe malaria with parasitemia below 10% and a clinical description associated with experimental cerebral malaria (ECM), including convulsion and paralysis of the hind legs, as well as dorsal deformation. The other half died from mild parasitemia (from 16% to 46%).

### 3.2. AQ 5 mg/kg Group (Figure 2 and Figure 3)

In the AQ 5 mg/kg group, 62.5% of mice died between D15 and D16 with parasitemia below 10% and with more severe signs of ECM than those in the CTL group. A total of 12.5% of mice died on D20 with higher parasitemia (24%) associated with rising anemia but also with a mild clinical schedule associated with experimental cerebral malaria (ECM). Only two mice stayed alive until D35, with low parasitemia (<0.2%).

### 3.3. MQ 5 mg/kg Group (Figure 2 and Figure 3)

In the MQ 5 mg/kg group, 87.5% of mice died on the same day on D18. Diagnosis revealed a critical status for ECM with a high score for the alteration of autonomous function, alteration of muscle tone and strength. The mice exhibited coordination disorder, ataxia, paralysis of the right front limb (three mice) and strongly modified behaviour. Only one mouse stayed alive until D35, with low parasitemia (<0.2%).

### 3.4. MB 10 mg/kg Group (Figure 2 and Figure 3)

In the MB 10 mg/kg group, 75% of mice died between D13 and D16. They experienced a severe form of ECM with convulsions and an opisthotonos-like stance and low parasitemia ranging from 2% to 10%. Ataxia and paralysis were also diagnosed in three mice. In total, 25% mice died later on D27 with severe anemia and high parasitemia (>70%). Diagnosis revealed splenomegaly and multi-visceral lesions.

### 3.5. MB with 10 mg/kg for Five Days Associated with AQ 5 mg/kg for Three Days (AQMB) Group (Figure 2 and Figure 3)

In the group receiving AQ plus MB combination therapy (AQMB), no mice died. Moreover, none of the mice exhibited an abnormal posture associated with lesions that impair brain and muscle function commonly observed in ECM. Furthermore, parasitemia did not exceed an average of 0.1% after the end of the curative injection.

### 3.6. MB with 10 mg/kg for Five Days Associated with MQ 5 mg/kg for Three Days (AQMB) Group (Figure 2 and Figure 3)

In the group receiving MQ plus MB combination therapy (MQMB), 37.5% of mice died between D19 and D26 with parasitemia ranging from 6% to 14.2%. They exposed a partial or complete clinical schedule for ECM with paralysis of at least two limbs, head deviation, pilo-erection, urinary retention and a rigid abdomen (revealing a lack of faecal elimination). The respiratory rate was also impaired (one mouse), suggesting potential acute respiratory distress syndrome (ARDS), but we were not able to investigate this hypothesis further. Meanwhile, five mice stayed alive until the end of the protocol (D35) with parasitemia under 0.01%. One mouse died after D35, and on D42 with high parasitemia (85%).

### 3.7. Comparison between the Different Treatments

All treatments demonstrate a significant effect (*p* < 0.0001; Mantel-Cox test and *p* < 0.0001; Logrank test) in comparison with non-treated mice. Every single therapeutic group, AQ (*p* < 0.0001), MQ (*p* < 0.0001) and MB (*p* < 0.0001), showed an increased survival rate in comparison with the CTL group, although neurological injuries and clinical follow-up highlight a form of cerebral malaria in AQ, MQ and MB. In the MQMB group, the survival rate was also significantly higher in comparison with the CTL group (*p* < 0.0001) but MQMB treatment offered a partial reduction or prevention of cerebral malaria. The survival rate with MQMB was significantly increased in comparison to MQ (*p* = 0.0079) and MB (*p* = 0.0039).

The best therapeutic group was the AQMB combination group where the survival rate was significantly increased (*p* < 0.0001) in comparison with the CTL group. The survival rate was also significantly different between AQMB and AQ (*p* = 0.0024) but also between AQMB and MB (*p* < 0.0001). Meanwhile, we found no statistical difference in the survival rate between AQMB and MQMB (Figure 2).

The main point of interest is the evolution of parasitemia (Figure 3). We found a significant difference when we compared the evolution of all parasitemia levels (*p* < 0.0001, ANOVA and Kruskal–Wallis test). Using Welch’s *t* test, we found significant differences between MB and CTL (*p* = 0.0415), MB and AQ (*p* = 0.0155), but also between MB and MQ (*p* = 0.0141). The combination therapies AQMB and MQMB were also found to be significantly different in comparison with the MB group (*p* = 0.0127 and *p* = 0.0395), respectively. Meanwhile, the parasitemia level for the MQMB group was also found to be significantly different in comparison with AQ (*p* = 0.0282), MQ (*p* = 0.0167) and AQMB (*p* = 0.0086). There was no statistical difference in other groups.

**Figure 4:**
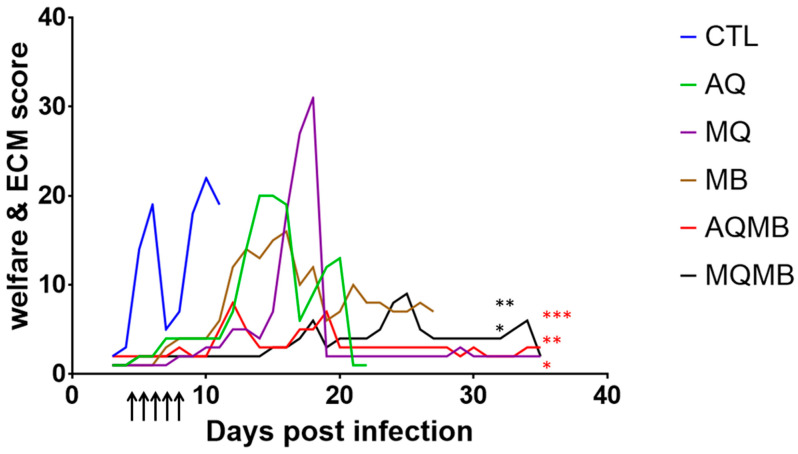
ECM score evolution. The ECM score was evaluated from D3 to D35 for all therapeutic groups. In blue, Control (CTL) with saline solution for five days. In green, AQ 5 mg/kg for three days. In purple, MQ with 5 mg/kg for three days, In brown, MB with 10 mg/kg for five days. In red, MB with 10 mg/kg for five days associated with AQ 5 mg/kg for three days (AQMB). In black, MB with 10 mg/kg for five days associated with MQ 5 mg/kg for three days (MQMB). Black arrows indicate days of treatment. A score below 4 indicates normal clinical status for mice. * = significant versus CTL, ** = significant versus MB, *** = significant versus AQ. Asterisk in red for AQMB and in black for MQMB.

To complete our work (Figure 4), we designed our score grid based on the literature in order to assess not just the welfare of the mice but also ECM [47,48,49]. The AQMB group (*p* = 0.0091) and MQMB group (*p* = 0.0112) demonstrated a significant difference compared with the CTL group. A significant difference was shown for the AQMB group compared with the AQ group (*p* = 0.0108) but also with the MB group (*p* = 0.0001). Meanwhile, in the MQMB group, we confirmed the survival results. The MQMB group demonstrated a significant difference (*p* = 0.0003) in comparison with the MB group but we did not find a statistical difference between the MQMB and MQ groups (*p* = 0.47).

## 4. Discussion

This study contributes additional data about MB and its use for preventing cerebral malaria in a malaria therapeutic rodent model. We designed this experiment considering the issues that malaria management represents.

We therefore used an experimental model in which mice died with neurological symptoms between D6 and D12 post-infection, as a reliable model to confirm our hypothesis.

AQ (5 mg/kg for three days) and MQ (5 mg/kg for three days) are efficient at reducing parasitemia and protecting mice as long as the treatment is present in the bloodstream of mice infected with *P. berghei* ANKA. However, these two drugs used alone did not provide full protection against cerebral malaria in this model once they had been eliminated from the blood stream.

We observed the same features in the clinical follow-up for mice treated with MB (10 mg/kg for five days), although 25% of the mice survived beyond D12 (experimental cerebral malaria step) but died with parasitemia over 70%. MB was efficient in decreasing parasitemia and providing protection given its half-life in the bloodstream [64]. Our data are complementary and do bring our previous analysis into question [33,35], but underline the importance of the interval between diagnosis and adoption of the therapeutic approach. Indeed, we previously showed that 78% of mice treated with 10 mg/kg MB alone survived and presented no specific signs of cerebral malaria or detectable parasites [33,35], whereas in this study, 75% of mice died between D13 and D16 with ECM symptoms and parasitemia ranging from 2 to 10%. In the previous studies, the mice were infected with 10^5^ parasitized erythrocytes and treated when the parasitemia reached 0.1% by daily MB ip injection, whereas in this study, the mice were infected with 10^6^ parasitized erythrocytes and treated when the parasitemia reached at least 1%. These data suggest that MB alone cannot control the parasitemia and cannot prevent or treat cerebral malaria once the disease is well established. In MB (10 mg/kg for five days) associated with AQ (5 mg/kg for three days) (AQMB), we demonstrate full protection with controlled parasitemia and no cerebral signs according to our score grid. No mice died in the AQMB group and they remained alive after the end of our protocol (D35), with the same clinical status. In the MB (10 mg/kg for five days) associated with the MQ group (5 mg/kg for three days) (MQMB), we observed only partial protection. Three mice died with low parasitemia ranging from 6% to 14.2% and exhibited a partial or full clinical schedule related to ECM. However, one mouse, which was still alive at the end of our protocol, exhibited high parasitemia (80%). By comparing both MB-based combination therapies, no significant difference was found in the survival rate, although we observed a significant difference in the evolution of parasitemia. Our ECM score grid suggests that in the AQMB and MQMB groups, treatment decreased the cerebral symptoms related to ECM. Our evaluation grid also suggests that even if AQ, MQ and MB increased the survival rate, mice in these groups presented modifications in their behaviour and clinical schedule.

Interestingly, no mice treated with AQMB died, whereas 37.5% of those treated with MQMB died. This may be explained by the mechanism of MB, but also a full potentiation between MB and AQ, and a partial synergy between MB and MQ. This last point would demonstrate a slight difference between our in vitro and in vivo studies. The combination of MB plus AQ has been demonstrated to be highly efficient in treating uncomplicated malaria in children from Burkina Faso [65].

A limitation of this study is that it is difficult to translate in vivo study results in animal models into actual clinical treatment in patients. In vivo pre-clinical studies allow for evaluation of in vivo drug activity, pharmacodynamics and pharmacokinetics and toxicities. However, the results obtained may vary between humans and other species [66]. A dose of 500 mg of methylene blue led to an average maximum concentration (C_max_) of 1600 ng/mL in plasma and an elimination half-life time (t_1/2_) of 7.2 h in humans [67]. An oral dose of 45 mg/kg of methylene blue led to half-life times in the range 4–5 h [64]. An oral dose of 400 mg of amodiaquine in humans led to an average C_max_ of 26 ng/mL and t_1/2_ of 7.1 h in plasma [68], whereas a dose of 1 mg/kg administered to mice led to t_1/2_ of 7.0 h [69]. An oral dose of 15 mg/kg of mefloquine administered in humans led to an average C_max_ of 2820 ng/mL and t_1/2_ of 346 h in whole blood [70]. The t_1/2_ was estimated around 16 h after an oral dose of 10 mg/kg in mouse [71]. A more reliable and predictive transition from mice to humans can be improved by the use of a *P. falciparum* humanized mouse model where efficacy results closely match the results in infected humans [72].

Concerning the mechanisms of MB, it exhibits an intrinsic inhibition of heme polymerization within the food vacuole [73]. Moreover, MB also targeted the *P. falciparum* glutathione reductase [74].

MB gives us a new hope in the fight against malaria but also with regard to the strengthening of malaria resistance by the prevention of methemoglobinemia, a serious complication of malarial anemia [75]. Its ability to supplement current chemotherapy reveals that this molecule could be an efficient tool to prevent and decrease cerebral malaria, even in late therapeutic approaches. The combination of MB plus artemisinin derivatives has already been found to exert synergistic in vitro activity [36] against *P. falciparum* and to prevent cerebral malaria and deaths due to malaria in a cerebral malaria murine model [33]. Moreover, the combination of standard methylene blue and artesunate was more effective by *intravenous* administration than either methylene blue or artesunate alone in a rat model infected by *P. berghei* and in models of *P. cynomolgi*-infected rhesus monkey [76]. The triple drug combination, including MB, AQ and artemisinin derivatives, was effective against *P. falciparum* gametocytes [30,77]. Moreover, MB significantly increased the in vitro plasmodial schizontocidal activity of ASAQ (artesunate plus amodiaquine) [78]. This triple combination would be a good candidate for treating artemisinin-resistant malaria and reduce malaria transmission.

## 5. Conclusions

Current standard antimalarial drugs such as AQ and MQ were unable to prevent cerebral malaria in our mouse model after their elimination from the bloodstream. This experiment underlines the importance of the interval between diagnosis and treatment with common antimalarial drugs, even with MB alone, which failed in our protocol to prevent death from cerebral malaria. However, 100% of the infected mice treated with MB in combination with AQ were protected from cerebral malaria. Moreover, our previous data, supplemented by this study, highlight the efficacy of MB as a complementary treatment to prevent malaria. Faced with the spread of resistance, a triple drug combination, including MB, AQ and artemisinin analogue, has the potential to treat resistant malaria and reduce malaria transmission.

## Data Availability

The datasets analysed in this study are available from the corresponding author on reasonable request.
